# Collectanea

**Published:** 1849-01

**Authors:** 


### Collectanea.

*On some Points in the Treatment of Hare-Lip.*—[Mr. Walton's observations refer to that form of hare-lip which is accompanied by cleft maxilla, one of the edges of the divided alveolus projecting. Respecting the treatment of these cases, he says :]

The usual practice is to excise the bit; and from what I have read and seen, I know that the bone-forceps have at times been freely applied. But obviously a great ill is incurred by the loss of bone from a part where so many and such important services are required of it. It is then to save and make available this piece of bone, which would otherwise be useless, and at the same time an obstacle, that I write.

During the last winter Mr. Fergusson was kind enough to allow me the opportunity of seeing him operate for a double hare-lip, involving the maxilla on each side; and Gensoul's method of bending back the central portion of bone was resorted to. Shortly after I availed myself of the information I had gained; for three cases of hare-lip were brought to me within a few weeks of each other, each with labial and maxillary clefts, two with entire division of hard and soft palate, one with the alveolus only divided. In all the protruding bone was very prominent, and the deformities bore a great resemblance to each other. These did not admit of Gensoul's method, but with a slight alteration I was enabled to apply his principle.

I shall not uselessly go into the steps of operating on the lips; enough to say that it is effected with a small scalpel and my fingers, and that I detach the soft parts from the subjacent bones more extensively than is generally done. I now cut through the protruding alveolus in its entire thickness, applying the bone-forceps at the spot about corresponding to the space between the first and second incisores, (and it has happened in all the cases that the deviation from the natural contour commenced just about there,) and bend back to the desired level the partially detached portion, which, I may remark, contains the rudiments of the two front teeth.

Dividing the alveolus at the part described may not be without benefit, since probably it interferes as little as it is possible with the future teeth. The soft parts can now be brought together with as much facility as when only fissure of the lip exists. Not much force is required in thus dealing with the bone after the forceps have been applied. There is no fracture, but a yielding, which I imagine may be ascribed to the yet imperfectly ossified incisor and palatine portions of the maxilla. I retain the lip *in situ* with pins and the twisted suture, according to the ordinary method. Of the after treatment I have no remarks to offer. There remained, in one of the cases, an irregularity at the free margin of the alveolus, the depressed portion descending a little below the natural line; but I did not deem any interference with it necessary.

Authorities differ about the age when the operation should be performed; the majority advise a late operation, some preferring the age of two years, some even later than that. If a portion of bone is to be removed, I do not think it matters much about the delay; but I hold it to be necessary to ensure success with the plan I recommend, that the operation should be resorted to at some period before dentition; and I should say that after the child is a month old the sooner it is done the better. One reason assigned for delay is, that very young children cannot bear the shock and loss of blood consequent on the operation. With modesty I would say that I believe the fear to be greatly exaggerated. We know that infants undergo more severe operations with success. On this subject Mr. Fergusson says, "From all my reflections and experience on the question, I am more than ever disposed to recommend a very early operation. Within the last twelve months I have operated on five infants, all of them under three months, with the most satisfactory results; and these cases, with others which I have previously had, are sufficient to induce me to pursue a similar practice in all instances of the kind which may come under my notice, unless there be some apparent indication not to interfere." And again, "I once asked the late Dr. Abercrombie, of Edinburgh, the result of his experience on this point," i. e. convulsions ensuing in children while undergoing operations, "and he could not bring a single instance to his recollection, where convulsions could be fairly attributed to an operation." Many instances have been recorded of infants of only a few days old having been operated on for hare-lip with success: other objections I shall not answer. The respective ages of my little patients were four, six and eight weeks. All were unusually small and thin, one peculiarly so. I have operated on children under six months, and with good results. In the case with the alveolus only divided, and the palate not implicated, there is not the slightest aperture remaining in the bone, for the piece pressed back accurately filled the chasm. The others are less perfect, but the little gap that exists is very trifling.

It remains for me to say that the result of my cases has been fully satisfactory. When similar affections come under my care, I shall use the same means, and not, as I have in other instances, remove any portion of bone.—*Medical Times.*—*Braithwaite's Retrospect.*

---

*Notice to Manufacturers.*—At the last annual meeting of the Mississippi Valley Association of Dental Surgeons, the following resolution was passed, and the Corresponding Secretary directed to communicate it to the different manufacturers throughout the United States:

*Resolved,* That this society offer to the manufacturer a gold medal worth twenty dollars, for one hundred of the best teeth offered, including specimens of gum teeth, of molares, bicuspidati, and common plate and pivot teeth in equal proportions; and that for the second best, of the same amount and character, be presented a silver medal worth ten dollars.

The teeth may be sent to care of Corresponding Secretary, who will lay them before the Committee, and the manufacturer's name will not be made known until after suitable tests have been made and the premiums awarded. The lots not receiving premium, will be subject to the order of the senders; and no names will be announced but of those who receive premiums. We hope for a lively competition.

The teeth must be received before the first of Sept. 1849.

E. Taylor, Cor. Sec.

[*Dental Register.*

---

*On the use of Collodion in Asbestos in stopping teeth.*—Sir:—Since the invaluable paper on collodion, by Mr. Erasmus Wilson, which appeared in your journal in November last, I have frequently applied collodion in severe cases of tooth-ache, arising from exposure of the nerve, with perfect success, when no persuasion could induce the patient to submit to extraction, either with or without the use of chloroform or ether. The method I adopt is, to let the patient first wash the mouth with warm water, in which a few grains of bicarbonate of soda have been dissolved. I then remove from the cavity any foreign substance likely to cause irritation. After drying the cavity, I drop from a point the collodion, to which have been added a few grains of morphia, after which I fill the cavity with asbestos, and saturate with collodion. Lastly, over this I place a pledget of bibulous paper. In a few seconds the whole becomes solidified, and forms an excellent non-conducter of heat and cold to the exposed nerve. By occasionally renewing this I have been enabled to effect a more durable stopping with gold leaf.

I remain, your obedient servant, J. Robinson.

[*London Lancet.*

*Lawrence's Improved Portable Blow-pipe.*—I have just received from Mr. Henry Lawrence, now temporarily resident in Allentown, Pa., a most beautiful little bellows blow-pipe, recently invented by him, which, for the perfection of its construction, the ease and facility with which it is used, its extreme portability, and though last, not least, its great cheapness compared with all others now in use, cannot, I think, fail highly to recommend it to dentists, and to any other persons whose occupation requires the use of such an instrument.

The whole machine is but about nine inches long, eight inches wide, and six inches deep; and even these small dimensions may be contracted if greater portability be desired, by removing certain portions of it; for every part of the blow-pipe, excepting the bellows, may be taken in pieces and reunited in one minute's time.

It is, in brief, constructed as follows: A small double bellows is made, with a treadle for the foot fixed horizontally over it, to one end of which a hinge is attached, the other being rendered stationary by a little hasp and staple. Instead of weights, the bellows is made to rise and fall upon the application of the foot to the treadle, by two springs of coiled brass wire, properly attached to the machine. The air passes through a long flexible tube, with a brass jet attached, by means of which the operator obtains a facility in the management of the flame, which, in my opinion, alone renders this little instrument superior to any I have yet seen.

I feel assured, Messrs. Editors, that few dentists who have hitherto contented themselves with the common mouth blow-pipe, will fail to supply their laboratories with this simple and most effectual little machine, after they shall have witnessed the ease and certainty with which it performs its appropriate work. Its price, also, which I presume will not exceed one-third that of the ordinary bench blow-pipe, taken in connexion with the superiority of its operation, must, I think, speedily induce a large majority of our dentists to adopt it so soon as it shall be offered for sale.

[*Dental News Letter.*

---

